# Persistent Postconcussive Symptoms in Children and Adolescents

**DOI:** 10.15844/pedneurbriefs-32-11

**Published:** 2018-10-02

**Authors:** Zarraf Arefeen, Syed Mosab Kazmi, Safiullah Shareef

**Affiliations:** 1 Department of Neuroscience, University of Texas Dallas, Richardson, Texas; 2 Texas Child Neurology, Plano, Texas

**Keywords:** Concussion, Physical Activity, Traumatic Brain Injury

## Abstract

Investigators from the Children’s Hospital of Eastern Ontario, Boston Children’s Hospital, Alberta Children’s Hospital, University of Montreal, McGill University Health Center, Hospital for Sick Children, University of Calgary, and the University of Ottawa researched the association between early physical activity and persistent postconcussive symptoms (PPCS).

Investigators from the Children’s Hospital of Eastern Ontario, Boston Children’s Hospital, Alberta Children’s Hospital, University of Montreal, McGill University Health Center, Hospital for Sick Children, University of Calgary, and the University of Ottawa researched the association between early physical activity and persistent postconcussive symptoms (PPCS). These researchers conducted a prospective study of 3063 patient aged 5.00 – 17.99 years with acute concussion, meeting concussion diagnosis criteria according to the 2012 Zurich consensus statement. Physical activity participation and postconcussive symptom severity were rated using standardized sport concussion questionnaires conducted in the ED and at days 7 and 28 post-injury.

Of the 2413 patients who provided full data, 733 (30.4%) met criteria for PPCS. At 7 days enrollment, 1677 (69.5%) patients reported participating in some physical activity. This included predominantly light aerobic exercise, but also sport specific exercise, non-contact training drills, full contact practice, and return to competition. 736 (30.5%) patients reported no physical activity in the first 7 days post-concussion. Of the 1677 patients who engaged in early physical activity, 523 (31.3%) were symptom free at day 7 and 803 (48.0%) had at least 3 persistent or worsening postconcussive symptoms at day 7. Of the 736 patients who denied participating in early physical activity, 584 (79.5%) had at least 3 persistent or worsening postconcussive symptoms at day 7.

To account for potential confounding variables, propensity scores were developed and utilized in the statistical analysis. Across various analytic approaches, the researchers consistently found that early return to physical activity was associated with a lower risk of PPCS as compared with no physical activity [1,2].

COMMENTARY. Approximately one-third of children after having an acute concussion experience ongoing somatic, cognitive, and psychological or behavioral symptoms, referred to as PPCS [3]. In 2012, a Zurich consensus statement from the 4th International Conference on Concussion in Sport recommended a graduated return to play protocol with an initial stage of no activity as a means of reducing PPCS. However their recommendations were not based on empirical data. In contrast, this prospective study concludes that early activity is correlated with a decrease in PPCS. Although the early physical activity cohort was divided into subcategories of activity intensity, the study could not conclude its relevance. Other research with smaller cohorts have indicated that high activity levels may worsen neurocognitive recovery [4].

The data could not differentiate what factors would contribute to higher early physical activity participation. For example, a patient who has less symptoms might be more likely to participate in increased activity as opposed to a patient with greater symptoms at onset of the concussion. Based on the study design, the data also could not indicate the frequency and the exact onset of the participants’ physical activity. Further research should be considered to determine the optimal dosing of early activity to reduce PPCS. These issues have been discussed in the 5th International Conference on Concussion in Sport although have yet to be thoroughly studied [5].

## Disclosures

The author(s) have declared that no competing interests exist.
